# Supplementary material for: Phytochrome A is required for light‐inhibited germination of Aethionema arabicum seed
Source: New Phytol. 2025 Jul 1;247(5):2134–46. doi: 10.1111/nph.70344 (PMC12329165; doi:10.1111/nph.70344)
Supplement: Supplementary file 1 — Table S1 List of primers used for quantitative RT‐PCR analysis. Table S2 Cosegregation of long hypocotyl phenotype under far‐red with the mutation identified in koy2. Please note: Wiley is not responsible for the content or functionality of any Supporting Information supplied by the authors. Any queries (other than missing material) should be directed to the New Phytologist Central Office. [file NPH-247-2134-s001.pdf]

New Phytologist **Supporting Information**

Article title: **Phytochrome A is required for light-inhibited germination of *Aethionema arabicum* seed**

Authors: Zsuzsanna Mérai, Fei Xu, Anita Hajdu, László Kozma-Bognár, Liam Dolan

Article acceptance date: 20 May 2025

The following Supportive Information is available for this article:

Table S1: List of primers used for quantitative RT-PCR analysis.
Table S2: Cosegregation of long hypocotyl phenotype under far-red with the mutation identified in *koy2*.

Supporting Table S1 List of primers used for quantitative RT-PCR analysis.

Name	Nucleotide sequence	Accession number v3.1
AearUBQ10_for	GAGGATGGCCGAACATTG	*Aa3LG9G835 (v3.0)*
AearUBQ10_rev	TGCCCGTTAGGGTTTTGA	
AearAPC2_for	TCTCCTGCAATCGAGGACTT	*Aa31LG10G13720*
AearAPC2_rev	GCAGTGAGCAACCGGTATTT	
AearCHS_for	GGCTCAAAGAGCTGATGGTC	*Aa31LG5G11220*
AearCHS_rev	TCGGTCATGTGGTCACTGTT	
AearPHYA_for	GGAGAAGTCTTCGGGACACA	*Aa31LG1G5460*
AearPHYA_rev	TTTCTCCGGTTCTTGACTGG	
AearGA3ox1_for	TCTTCGTCACCTCCCTGACT	*Aa31LG7G270*
AearGA3ox1_rev	GATGAGCGGGAGAGTTGTGT	
AearGA2ox3_for	CGCGTCTCTCTTAACCCAAC	*Aa31LG10G6850*
AearGA2ox3_rev	TCACATGCCTTGACCATTTG	
AearNCED5_for	GCCGTTTGATCTTGACGCTC	*Aa31LG6G5570*
AearNCED5_rev	ACGGAGTTTAGTTTACGGCGT	
AearNCED6_for	GCTTCTTCAGCTCTCGACAA	*Aa31LG8G10550*
AearNCED6_rev	GAACCGTTGGATCAGTCGGT	
AearCYP707A2_for	GCGGTTCCAACAAAGAAAAC	*Aa31LG5G7160*
AearCYP707A2_rev	GAGTGGCGAAGAAGGAATTG	

Supporting Table S2. Cosegregation of long hypocotyl phenotype under far-red with the mutation identified in *koy2*.

♀ *koy-2* x ♂ WT			
Phenotype	Number	Genotype	Number
Long hypocotyl	11	*koy2/koy2*	11 (100%)
		WT/*koy2*	0
		WT/WT	0
Short hypocotyl	47	*koy2/koy2*	0
		WT/*koy2*	35 (60.3%)
		WT/WT	12 (20.7%)
